# The price of curing cancer

**DOI:** 10.1186/s12913-021-07327-x

**Published:** 2021-12-11

**Authors:** Afschin Gandjour

**Affiliations:** Frankfurt School of Finance & Management, Adickesallee 32-34, 60322 Frankfurt am Main, Germany

**Keywords:** Cancer, Cure, Health expenditure

## Abstract

**Background:**

Health care systems around the world struggle with high prices for new cancer drugs. The purpose of this study was to conduct a *gedankenexperiment* and calculate how much health expenditures would change if a cure for cancer through pharmaceutical treatment were made available. The cancer cure was conceived to eliminate both cancer deaths and the underlying morbidity burden of cancer. Furthermore, the cure was hypothesized to arrive in incremental steps but at infinitesimally small time intervals (resulting, effectively, in an immediate cure).

**Methods:**

The analysis used secondary data and was conducted from the viewpoint of the German social health insurance. As its underlying method, it used a cause-elimination life-table approach. To account for the age distribution of the population, the study weighted age-specific increases in remaining life expectancy by age-specific population sizes. It considered drug acquisition costs as well as savings and life extension costs from eliminating cancer. All cancer drugs that underwent a mandatory early benefit assessment in Germany between 2011 and 2015/16 and were granted an added benefit were included. Data on age- and gender-specific probabilities of survival, population sizes, causes of death, and health expenditures, as well as data on cancer costs were taken from the German Federal Office of Statistics and the German Federal Social Insurance Office.

**Results:**

Based on the cause-elimination life-table approach and accounting for the age structure of the German population, curing cancer in Germany yields an increase in average remaining life expectancy by 2.66 life years. Based on the current incremental cost-effectiveness ratio of new cancer drugs, which is on average €101,493 per life year gained (€39,751/0.39 life years), the German social health insurance would need to pay €280,497 per insuree to eliminate cancer. Dividing this figure by current average remaining lifetime health expenditures yields a ratio of 2.07, which represents a multiplier of current health expenditures.

**Conclusions:**

Eliminating cancer at current price levels would more than triple total health expenditures in Germany. As the current price of a cure requires a drastic reduction of non-health consumption, it appears that current prices for cancer drugs already on the market (i.e., small steps towards a cure) need careful reconsideration.

**Supplementary Information:**

The online version contains supplementary material available at 10.1186/s12913-021-07327-x.

## Key points

- The purpose of this study was to conduct a *gedankenexperiment* and calculate how much health expenditures would change if a cure for cancer through pharmaceutical treatment were made available.
- The study shows that eliminating cancer at current price levels would more than triple total health expenditures in Germany.

## Introduction

Health care systems around the world struggle with high prices for new, innovative cancer drugs. In the U.S., for example, median annual costs of new cancer drugs are now above $150,000 [1]. They contribute to about one third of projected peak worldwide annual sales of new drugs [2]. From a manufacturer’s perspective, high prices are justified because largely fixed costs for research and development (R&D) need to be distributed over a small cancer patient population. In addition, high prices provide an incentive to continue to invest in R&D and obtain future innovations [3]. However, when setting launch prices of cancer drugs in the U.S. over a “typical duration of treatment” in relation to survival gains, the resulting cost-effectiveness ratios are now, on average, above $200,000 per life year gained [4]. Thus, ratios are higher than the willingness-to-pay threshold commonly applied in the U.S. academic literature, which is in the range of $50,000 to $100,000 per quality-adjusted life year (QALY) gained [5]. On the other hand, cancer drugs currently account for less than 20% of total drug costs in Western countries [6]. Hence, even dramatic increases in cancer drug costs will have little impact on total health expenditures in the foreseen future. At least in countries with generous public health insurance coverage total expenditures for cancer drugs thus still seem affordable. The contrast between high prices and relatively low budget impact has led to diverging recommendations by experts and policymakers in countries with generous public health insurance coverage, ranging from ‘wait and see’ to considerable price cuts [7]. In terms of affordability, the U.S. is somewhat of an outlier: although it spends less as a percentage of total drug costs than many other Western countries [6], affordability is more of an issue. The reason is that cancer drugs need to be covered to a considerable degree by private means, i.e., out-of-pocket payments [8,9].

The purpose of this study was to contribute to the ongoing debate on policy implications of high cancer drug prices. To this end, I conducted a *gedankenexperiment* (thought experiment) that envisioned in a deliberately chosen extreme-case scenario a cure for cancer through pharmaceutical treatment. I calculated how much health expenditures would change if such cure were made available. This cancer cure was conceived to eliminate both cancer deaths and the underlying morbidity burden of cancer as well as new cancers in cancer survivors. Furthermore, I hypothesized that a cancer cure would arrive in incremental steps (consistent with past technology diffusion) but at infinitesimally small time intervals (resulting, effectively, in an immediate cure). This follows the idea that small incremental gains in survival conferred by single drugs translate into large improvements in survival when drugs are given in combination [10,11].

I determined the price for a cure based on the current ‘exchange rate’ between money and health. This calculation was based on the following argument: If we are willing to pay, based on the current exchange rate, X euros for a small incremental survival gain, then we should be willing to pay a proportionally larger amount for the sum of all incremental survival gains. That is, if we are willing to pay X euros to increase survival by Y/n years, then we should be willing to pay n · X euros to increase survival by n · Y/n years. Conversely, if we are willing to pay Z euros to increase survival by m years, then we should be willing to pay (no more than) Z/n euros to increase survival by m/n years. These calculations assume i) the absence of a budget for health care expenditures, ii) an extra-welfarist perspective, which is commonly interpreted to include health (e.g., life years) in its evaluation space but to exclude the utility of life years (in contrast, adopting a welfarist perspective would require assuming a constant marginal benefit of life years), and iii) profit-maximizing behavior of pharmaceutical manufacturers.

Needless to say that our scenario of an immediate cure is unrealistic: When extrapolating the mortality decline over the past 20 years [12] into the future, it would take approximately 44 years for a final cure to arrive and obtain a normal remaining life expectancy for cancer patients (at present, the 5-year mortality rate of cancer patients compared to the normal U.S. population is increased by 31% [12]). Nevertheless, the scenario of an immediate cure is useful in order to analyze whether current drug prices are justified. Likewise, the absence of budget restrictions cannot be considered realistic. Yet, assuming it is helpful in laying out the consequences of spending on cancer care at current prices.

## Methods

### Life expectancy

In order to determine gains in life expectancy from a cancer cure I used a cause-elimination life-table approach [13–15]. A cause-elimination life table addresses the hypothetical question of what a life table cohort’s mortality experience would be if a particular cause of death (here: cancer) were eliminated. That is, standard life table functions are re-estimated assuming that no one dies from the selected cause of death. Assuming that deaths occur, on average, halfway at each age, I applied the so-called life-table method [16] to life years (and costs). In order to account for the age distribution of the population, I determined remaining life expectancy with and without cancer elimination at each age and weighted age-specific gains in remaining life expectancy by age-specific population sizes (see Appendix for details). Thus, I calculated the average gain in life expectancy in the total population.

### Costs

The analysis was conducted from the viewpoint of the German social health insurance (SHI). Incremental costs of a cancer cure can be divided into the following components cf. [17]: i) incremental drug costs including acquisition costs (i.e., pharmacy retail prices net of mandatory rebates for the SHI), costs of adverse events (AEs), and costs of drug-related services such as application, counseling, and monitoring; ii) incremental savings from eliminating cancer morbidity (AEs are not included); and iii) life extension costs from eliminating cancer mortality.

In our scenario, the third cost component, i.e., life-extension costs, is unrelated to cancer because cancer is eliminated. To determine life-extension costs, I multiplied the cumulative probability of an individual at age *i* of surviving until age *j* (i.e., the product of age-specific survival probabilities up to age *j*) as obtained from the life table with health expenditures at age *j* (see Appendix for a formalization). I took the sum over all ages *j* (thus obtaining the remaining health care expenditures of an individual at age *i*) and determined the difference between the cancer elimination scenario and the status quo, thus calculating life extension costs. To account for the age distribution of the population, I weighted age-specific life-extension costs by age-specific population sizes. I performed all calculations for men and women separately and then aggregated results.

In order to determine the cost of the drug treatment itself, I determined the current ‘exchange rate’ between money and health by dividing current incremental costs of new cancer drugs by the incremental survival benefit. By multiplying this ratio with the gain in life expectancy from cancer elimination (adjusted for the age distribution of the population as described above), I obtained the per-person (or per insuree) drug acquisition cost of curing cancer in the general population. Finally, in order to arrive at the total cost or price of curing cancer, I subtracted the portion of health expenditures attributable to cancer from the lifetime cost (thus accounting for savings from eliminating cancer morbidity) and added life extension costs from eliminating cancer mortality.

In a sensitivity analysis I analyzed how a potential reduction of the incremental costs of new cancer drugs beyond the time frame of the study (2011 to 2016) would affect results. A reduction of the incremental costs may be the result of increasing prices of comparator drugs, thus giving less leeway to charge a price premium. On the other hand, negotiated discounts have not increased since 2016 [18] (p. 240), which would have been expected in the event of decreasing price premiums. Furthermore, a decline in the size of target populations, which has been observed over time [18] (p. XX), and is consistent with the concept of drug ‘orphanization’, may cancel out a potential price decline from more expensive pricing comparators.

In addition to calculating the per-person (or per insuree) price of curing cancer in the general population (as delineated above), I also determined the drug price per cancer patient treated. This calculation does not attribute total cancer drug spending to each insuree (as above) but to each cancer patient. To this end, the annual cancer incidence in Germany was taken into consideration.

### Discounting

In the base-case analysis, I did not discount costs and health benefits as the reported survival benefits from cancer treatment [19] were undiscounted as well. In a sensitivity analysis, I discounted both costs and effects at the official rate of 3% p. a. in Germany [20]. Thus, I accounted for differences in the timing of cancer treatment and cure. For example, for a young cohort cancer incidence and hence cancer cure is on average more distant than for an elderly cohort.

### Data

In Germany, annual treatment costs for new cancer drugs launched between 2011 and 2015 and granted an additional benefit by the German Federal Joint Committee are €65,854 on average (calculated from the data set underlying reference 21, which was obtained from the author). Average annual costs of comparators are €26,102, resulting in incremental costs of €39,751.

Information on the average incremental survival benefit was taken from an analysis of all anticancer drugs launched in Germany between 2011 and 2016 and granted an additional benefit by the German Federal Joint Committee until June 2016. The analysis shows a median incremental survival benefit of 4.7 months or 0.39 years [19]. This result is similar to what was found in an analysis of 58 anticancer drugs approved in the U.S. between 1995 and 2013, showing an average incremental survival benefit of 0.46 years [4]. However, in both analyses the incremental survival benefits are underestimated because they are restricted to the trial period; i.e., they are not extrapolated beyond the trial period (strictly speaking, this is the case only for 47 out of 58 drugs in the study by Howard et al.; see below for further discussion).

To calculate life years gained from curing cancer based on the cause-elimination life-table approach, I used data from the German Federal Office of Statistics (probabilities of survival, population size, and disease-specific causes of death by age and gender up to the age of 100 years) for the year 2014 [22–24]. To calculate the change in lifetime health care expenditures resulting from a gain in life expectancy, I took data on average SHI expenditures by age and gender up to the age of 100 years from the German Federal Social Insurance Office [25] for the year 2014 as well (copayments, administration costs, and costs of non-mandatory health care services are excluded). For the calculation of savings from eliminating cancer morbidity I used data on cancer costs by age group in 2015 from the German Federal Office of Statistics [26] (age-specific data were unavailable for other years). The annual incidence of cancer in the German population (482,470 men and women in 2013) was obtained from the Robert Koch Institute [27], a German federal government agency and research institute responsible for disease control and prevention.

### Supplementary analysis

In this analysis I accounted for the fact that a new innovative medicine faces generic competition after losing patent and regulatory data protection, resulting in a decrease of the patent-protected price and an improvement in cost-effectiveness. Nevertheless, for new cancer drugs the situation is more complex due to the significant share of biologics and orphan drugs. Biosimilar competition is predicted to lead to a smaller price reduction than generic drug competition but may not even take place due to the relatively high hurdle for regulatory approval. For orphan drugs, generic drug competition may not take place due to a relatively small market size and if it does, the timing may be difficult to predict due to possible extension of the period of exclusivity. Given the lack of solid empirical data on the decrease of cancer drug prices over a drug’s lifecycle inside and outside Germany and the resulting uncertainty, I present this analysis as a supplementary one. Using UK data, Hoyle [28] calculated the average incremental cost-effectiveness ratio (ICER) of a drug over its whole lifecycle including the period after loss of patent protection. The so-called “life-cycle correction factor” determines how this ICER compares to the ICER at launch in relative terms. Based on 440 new chemical entities launched between 1981 and 2007, this modelling study estimated the average lifetime to be 33 years. Assuming a 4% real decline in drug prices per year and future incident cohorts, Hoyle [28] calculated that the average ICER was 36% below the ICER at launch. As the ICER includes non-drugs costs (which are assumed to stay constant over the lifetime), the reduction in prices over drug lifetime must be larger than 36%. Hence, a 36% reduction estimate falls in-between the true reduction in prices of the drugs analyzed by Hoyle [28] and the presumably lower reduction for cancer drugs and thus may provide a reasonable compromise.

## Results

Based on the cause-elimination life-table approach curing cancer in Germany yields an increase in life expectancy at birth by 3.25 life years. The average gain in the total population is 2.66 years (see Table 1). The resulting increase in lifetime health expenditures in the total population is small, however (€10,028). The reason is that life extension costs from eliminating cancer mortality are almost offset by savings from eliminating cancer morbidity.

**Table 1:** Health expenditure and life years per person over remaining lifetime (without consideration of the cost of drug treatment)

	Health expenditure (€)	Life years	Incremental health expenditure (€)	Incremental life years
Base case				
Current care	135,303	39.11		
Cancer cure	145,331	41.77	10,028	2.66
3% discount rate				
Current care	54,798	18.11		
Cancer cure	59,317	19.14	4519	1.03

Nevertheless, when adding the cost of drug treatment, the picture changes completely. The current exchange rate between money and health is on average €101,493 per life year gained (€39,751/0.39 life years). Thus, we would need to pay on average €270,469 (€101,493 · 2.66) for the cancer cure itself in order to obtain the gains in life expectancy from cancer elimination (accounting for the age structure of the population). Subtracting savings from eliminating cancer morbidity and adding costs of life extension increases the total to €280,497. Dividing the latter figure by the current remaining lifetime health expenditures (again adjusted for the age structure of the population) yields a ratio of 2.07, which represents a multiplier of current health expenditures. The multiplier changes only little (to 1.99) after discounting of costs and life years but more substantially when accounting for generic/biosimilar entry (to 1.35). When accounting for a 25% reduction in the incremental costs of new drugs, the multiplier falls in-between the two estimates (1.57). Based on the annual cancer incidence in Germany, the drug cost per cancer patient treated and cured is €704,099 even accounting for generic/biosimilar entry.

## Discussion

This study shows that eliminating cancer at the current exchange rate between money and health would increase total health expenditures in Germany 3.07-fold or by 207% in the base-case analysis and 2.35-fold accounting for generic/biosimilar entry. The underlying gain in life expectancy from cancer elimination is in line with the results of other studies. For example, the gain for female and male newborns in the Netherlands was reported to be 3.6 and 4.1 years, respectively, based on data from 2009 [29], whereas in the U.S. the gain for newborns was estimated to lie between 2 and 3 years in the period between 2001 and 2008 [30].

Based on the *gedankenexperiment* the percentage of income spent on SHI in Germany would grow from currently 15.7% (which includes an average supplementary premium of 1.1% [31]) to 37% even considering generic/biosimilar entry. Disregarding the macroeconomic implications of such labor cost increase (e.g., in terms of competitiveness of German goods and products in the international market), the question appears whether the German population would support the necessary drastic reduction of non-health consumption. Also, the reduction of non-health consumption could reduce the survival benefit of eliminating cancer. This will happen if the negative health impact of spending less on nutrition, hygiene, better social conditions, and so forth outweighs any positive impact such as a reduction in the use of cars.

Even a 50% discount from current prices for new cancer drugs in conjunction with the consideration of generic/biosimilar entry would still imply that 27% of income in Germany is spent on health care. To reduce this share to let’s say 20% of income it would be necessary to command an 83% discount from current prices. This implies not only to bend the ‘price curve’ but a much more drastic reversal of the current trend of increasing drug prices. Hence, it may be fair to say that, taken to an extreme, the R&D cost argument as the fundamental justification for today’s prices does not align well with the presumed willingness to pay of the German SHI. It seems at least questionable that insurees would be willing to pay this amount for a cancer cure in order to account for R&D costs, once the portion of their income spent on health care has reached a certain threshold and significantly cuts into their non-health spending. But if the extrapolated price for a cure lacks justification as implied in this study, then it appears that current prices even for small steps towards the cure (the small gains in life expectancy) need reconsideration as well. One may invoke the notions of diminishing marginal benefit of additional life years and diminishing severity of cancer here, on the basis of which the willingness to pay for more distant steps towards the cure would be lower than for the initial steps. This stands in contrast to what is implied by the concept of diminishing marginal benefit of R&D, however, which is that later market entrants are justified in commanding higher prices. The latter principle thus suggests that current prices cannot be easily compensated by sufficiently large discounts for products entering the market later.

One may counterargue that such discounts may even be possible when envisioning a single-step cure because R&D costs of such a drug would be distributed over a large patient population. In fact, in a similar *gedankenexperiment* to this one, Bhattacharya et al. [32] assumed a single-step cure at a cost of just $10,000 per cancer patient, which was deliberately chosen to be optimistically low even at the time of their publication. But a single cure would, of course, deviate from the past history of small incremental gains in life expectancy, which the present study uses as a basis for its calculation in order to test the plausibility of the R&D cost argument. Hence, such a miracle drug seems unrealistic, at least when it comes to curing cancer as such (acknowledging that for specific types of cancer or patient subgroups a cure may be both conceivable and affordable). One may counterargue that obtaining any immediate cure – be it through a miracle drug or the sum of small incremental innovations – is unlikely and purely hypothetical. Therefore, the *gedankenexperiment* would fail. Yet, similar hypothetical scenarios and thought experiments are common in the health economics literature. Consider, e.g., the question posed by the time trade-off (TTO) questionnaire, which elicits quality-of-life weights and underlies one of the most common health-related quality-of-life questionnaires used in clinical research, the EuroQol five dimensions questionnaire (EQ-5D): The TTO questionnaire asks for the number of remaining life years one is willing to give up in order to be cured from, say, cancer. This is very similar to the trade-off raised by this article, viz., how much non-health consumption (in monetary terms) we as a society are willing to give up in order to be cured from cancer. That is, in both cases we capture a trade-off involving a hypothetical cure for cancer.

In addition, one may criticize the logic of taking high prices for small incremental innovations to an extreme. But again, such linear extrapolation is common in the health economics literature. For example, when eliciting the willingness to pay for a QALY in the general public by a survey, the estimate is obtained only for a fraction of a QALY in order to avoid hitting an income constraint [33,34]. The willingness-to-pay value is then extrapolated to match a full QALY [33,34]. Similarly, the calculation of the ICER extrapolates the cost of gaining less than one QALY to a full QALY using linear extrapolation. The only difference is that this study extrapolates to more than one unit of health outcome whereas the former approaches extrapolate to exactly one unit of health outcome.

Furthermore, one may counter that a growing economy would be able to accommodate future cancer drug expenditure increases. However, while price levels may be sustainable, their justification based on R&D costs fails as shown in the extreme-case scenario envisioned in this study. Hence, there is a difference between what we are *able* to pay on the one hand and what we are *willing* to pay considering the opportunity costs on the other hand.

As a word of caution, modeling studies such as this one are rarely perfect due to constraints of resources, time, and information availability. On the one hand, our model even underestimates the costs of a cancer cure because costs of drug-related AEs and drug-related services are ignored and costs of cancer treatment are limited to a period of 1 year. That is, I do not account for the fact that some cancers have a chronic course, thus mandating treatment for more than 1 year. Also, costs of curing cancer do not include a potential premium for eliminating anxiety associated with cancer (cf. [35]). Fully accounting for these aspects would increase the costs of a cancer cure and support the conclusions of this paper. Furthermore, cancer survivors are at increased risk for cardiovascular disease [36]. It remains to be investigated, however, whether an increase in expenditure for cardiovascular disease (and thus the costs of a cancer cure) is offset by less spending on non-cardiovascular disease due to earlier death. On the other hand, costs of a cancer cure are overestimated because separate modelling of expenditure data for survivors and decedents as opposed to using age-specific average cost data would decrease life extension costs associated with the elimination of cancer mortality cf. [37]. Also, the survival benefit is underestimated as it is confined to the trial period [18]. Therefore, the current exchange rate between money and health is overestimated and so is the cost of a cancer cure. Some of the biases mentioned in this and the previous paragraph may cancel out, however.

Arguably, a more comprehensive assessment of the health gain from cancer elimination could be obtained through the QALY metric, which combines survival with a valuation of health-related quality of life [38]. I did not calculate QALYs, however, due to a lack of aggregated data on cancer-related quality of life. If currently available treatments reduced the morbidity and mortality burden of cancer to the same degree, results would be exactly the same as for the calculation of life years (because each life year gained would be associated with a proportional quality-of-life improvement both for the current and the remaining burden reduction). Yet, if the impact of current cancer drugs were smaller on the morbidity burden, a cancer cure would result in even higher expenditure when using the QALY metric. The reason is that the remaining morbidity burden that would need to be eliminated by a cure would become larger, resulting in more QALYs gained by a cure and higher expenditures based on the fixed exchange rate between money and health. In any case, even the QALY metric is not able to fully capture an elimination of cancer-associated anxiety.

Transferability of the results from a German setting to other jurisdictions depends, among others, on how the population burden of cancer, costs of new cancer drugs, and health care expenditures as a percentage of income compare to Germany. Taking the U.S. as an example, spending on cancer drugs as a percentage of total drug expenditures is lower than in Germany (11.5% vs. 15.9%) [6] but health care expenditures as a percentage of Gross Domestic Product (17.2% vs, 11.3%) and incidence of malignant neoplasms (318 vs. 284 per 100,000) are higher [39]. Therefore, given that these differences cancel out to some degree, the results of this study may also apply to other jurisdictions such as the U.S.

## Conclusions

This article has scrutinized the justification of high cancer drug prices for small gains in life expectancy. When taking high prices for small gains in life expectancy to an extreme, they do not seem to be aligned with the presumed willingness to pay by German social health insurees. From this perspective current prices do not seem justifiable, while acknowledging that they are sustainable at least in the medium turn, presuming that economic growth in Germany will return to pre-pandemic levels.

As stated in the introduction, high prices of new cancer drugs may not only be seen as a manufacturer’s compensation for past R&D but also as an incentive for future R&D. If the current exchange rate between money and health were decreased by stricter price control, would it disincentivize cancer research? The relationship between pharmaceutical sales and innovation is complex and while there is evidence for a positive effect of pharmaceutical sales on the number of clinical trials conducted at a national level, basic research activities may not be affected, at least not in the U.S. [40,41]. As the German market is much smaller in size, the impact of stricter price control on innovation is expected to be even less tangible. Nevertheless, stricter price control by payers will not be a straightforward panacea in face of potential market entry delays or even market withdrawals.

As an alternative solution to incentivizing R&D of future cancer drugs, it may be more efficient, from a policy perspective, to reallocate funds to preventive oncology. In fact, increasing prices for new cancer drugs makes prevention more cost-effective due to larger savings from avoiding cancer. Nevertheless, prevention may suffer from a problem analogous to that of a small target population, which is a potentially high number of patients that need to be enrolled in a prevention program to avoid one cancer death. In addition, the presence of life extensions costs from reducing cancer-associated mortality sets limits on efficiency gains. Needless to say, the cost-effectiveness of cancer prevention may also depend on the type of cancer. Therefore, the search for pragmatic solutions for the conundrum identified in this article needs to continue.

## Supplementary Information

**Additional file 1.**

## Data Availability

The datasets used and/or analyzed during the current study are available from the corresponding author on reasonable request.
