# Supplementary material for: The price of curing cancer
Source: BMC Health Serv Res. 2021 Dec 11;21:1328. doi: 10.1186/s12913-021-07327-x (PMC8665579; doi:10.1186/s12913-021-07327-x)
Supplement: Supplementary file 1 — Additional file 1. [file 12913_2021_7327_MOESM1_ESM.docx]

**Appendix**. Calculation of life expectancy gains and life-extension costs from cancer elimination in the general population.

Differences in life years ($\Delta LE$) and life-time costs ($\Delta C$) between cancer elimination ($y_{1}$) and no cancer elimination ($y_{2}$) are obtained in a population with individuals at age $i = 1,2,\ldots,x$ as follows:

$$LE\left( y \right)=\frac{\sum_{k=1}^{q} \left( \sum_{j=1}^{k} \left( \prod_{i=1}^{j} p_{i}(y) \right)\cdot N_{j} \right)}{\sum_{k=1}^{q} N_{k}}$$

, (A1)

$\Delta LY=LE\left( y=y_{1} \right)-LE(y=y_{2})$, (A2)

$EC\left( y \right)=\frac{\sum_{k=1}^{q} \left( \sum_{j=1}^{k} \left( \prod_{i=1}^{j} p_{i}(y) \right)\cdot N_{j}\cdot c_{j} \right)}{\sum_{k=1}^{q} N_{k}}$, (A3)

$\Delta C=EC\left( {y=y}_{1} \right)-EC({y=y}_{2})$, (A4)

where $LE$ denotes life expectancy, $EC$ is expected costs, $N_{j}$ denotes population size at age $j$, $p_{i}(y)$ denotes probability of surviving from age $i$ to $j$, and $c_{j}$ is costs incurred during time interval $(j, j+1)$. As shown, age-specific remaining life expectancy and health spending is weighted by age-specific population size. In equations A1 and A3 the denominator is total population size.
